# Systematic review with radiomics quality score of cholangiocarcinoma: an EuSoMII Radiomics Auditing Group Initiative

**DOI:** 10.1186/s13244-023-01365-1

**Published:** 2023-02-01

**Authors:** Roberto Cannella, Federica Vernuccio, Michail E. Klontzas, Andrea Ponsiglione, Ekaterina Petrash, Lorenzo Ugga, Daniel Pinto dos Santos, Renato Cuocolo

**Affiliations:** 1 Section of Radiology - Department of Biomedicine, Neuroscience and Advanced Diagnostics (BiND), University Hospital “Paolo Giaccone”, Via del Vespro 129, 90127 Palermo, Italy; 2 Department of Health Promotion, Mother and Child Care, Internal Medicine and Medical Specialties (PROMISE), University of Palermo, Via del Vespro, 129, 90127 Palermo, Italy; 3 Department of Radiology, University Hospital of Padova, Via Nicolò Giustiniani 2, 35128 Padua, Italy; 4 Department of Medical Imaging, University Hospital of Heraklion, 71110 Voutes, Crete, Greece; 5 Department of Radiology, School of Medicine, University of Crete, 71003 Heraklion, Crete, Greece; 6 Computational Biomedicine Laboratory, Institute of Computer Science, Foundation for Research and Technology, Vassilika Vouton, 70013 Crete, Greece; 7 Department of Advanced Biomedical Sciences, University of Naples “Federico II”, Via Sergio Pansini 5, 80131 Naples, Italy; 8 Radiology Department Research Institute of Children’s Oncology and Hematology, FSBI “National Medical Research Center of Oncology n.a. N.N. Blokhin” of Ministry of Health of RF, Kashirskoye Highway 24, Moscow, Russia; 9 IRA-Labs, Medical Department, Skolkovo, Bolshoi Boulevard, 30, Building 1, Moscow, Russia; 10 Department of Diagnostic and Interventional Radiology, Faculty of Medicine and University Hospital Cologne, University of Cologne, Kerpener Str. 62, 50937 Cologne, Germany; 11 Department of Radiology, University Hospital Frankfurt, Theodor-Stern-Kai 7, 60590 Frankfurt, Germany; 12 Department of Medicine, Surgery, and Dentistry, University of Salerno, Via Salvador Allende 43, 84081 Baronissi, SA Italy; 13 Augmented Reality for Health Monitoring Laboratory (ARHeMLab), Department of Electrical Engineering and Information Technology, University of Naples “Federico II”, Via Sergio Pansini 5, 80131 Naples, Italy

**Keywords:** Systematic review, Cholangiocarcinoma, Liver, Quality improvement

## Abstract

**Objectives:**

To systematically review current research applications of radiomics in patients with cholangiocarcinoma and to assess the quality of CT and MRI radiomics studies.

**Methods:**

A systematic search was conducted on PubMed/Medline, Web of Science, and Scopus databases to identify original studies assessing radiomics of cholangiocarcinoma on CT and/or MRI. Three readers with different experience levels independently assessed quality of the studies using the radiomics quality score (RQS). Subgroup analyses were performed according to journal type, year of publication, quartile and impact factor (from the Journal Citation Report database), type of cholangiocarcinoma, imaging modality, and number of patients.

**Results:**

A total of 38 original studies including 6242 patients (median 134 patients) were selected. The median RQS was 9 (corresponding to 25.0% of the total RQS; IQR 1–13) for reader 1, 8 (22.2%, IQR 3–12) for reader 2, and 10 (27.8%; IQR 5–14) for reader 3. The inter-reader agreement was good with an ICC of 0.75 (95% CI 0.62–0.85) for the total RQS. All studies were retrospective and none of them had phantom assessment, imaging at multiple time points, nor performed cost-effectiveness analysis. The RQS was significantly higher in studies published in journals with impact factor > 4 (median 11 vs. 4, *p* = 0.048 for reader 1) and including more than 100 patients (median 11.5 vs. 0.5, *p* < 0.001 for reader 1).

**Conclusions:**

Quality of radiomics studies on cholangiocarcinoma is insufficient based on the radiomics quality score. Future research should consider prospective studies with a standardized methodology, validation in multi-institutional external cohorts, and open science data.

**Supplementary Information:**

The online version contains supplementary material available at 10.1186/s13244-023-01365-1.

## Key points

- The quality of current radiomics studies on cholangiocarcinoma is insufficient, with a median radiomics quality score of 8–10, corresponding to 22–28% of the ideal quality score.
- None of the current studies conducted phantom assessment, imaging at multiple time points, prospective registration in a trial database, nor cost-effectiveness analysis.
- The inter-reader agreement of the radiomics quality score is good (ICC of 0.75; 95% CI 0.62–0.85) among readers with different levels of experience.

**Supplementary Information:**

The online version contains supplementary material available at 10.1186/s13244-023-01365-1.

## Introduction

Radiomics is a rapidly expanding area of active research with promising results based on the extraction and analysis of a large number of quantitative features from biomedical images [1]. Recent radiomics studies aimed to construct predictive models that can be combined with qualitative radiological features, clinical characteristics, and laboratory markers to develop decision support tools and improve patients’ care [1]. Several research studies proposed models based on computed tomography (CT) and magnetic resonance imaging (MRI) exams, with high performances for preoperative lesion characterization, prediction of treatment response, and assessment of prognosis after surgical resection [2]. Despite the promising results in research setting, there is still very limited translation in clinical practice due to the limitations of current radiomics research. These include heterogeneity of imaging acquisition protocols, segmentation, type of extracted features, and lack of validation in multicenter setting [3, 4]. Quality of radiomics studies represents a significant landmark for improvement of radiomics research and future clinical applications.

The radiomics quality score (RQS) has been proposed by Lambin et al. [5] for assessing the quality of radiomics studies based on 16 items related to the main steps of radiomics workflow. In the setting of liver imaging, recent systematic reviews have applied the RQS for assessment of quality of radiomics studies on hepatocellular carcinoma [6–9] and hepatic metastases [10] reporting an overall RQS of 8–14 (corresponding to 23–39% of the total score) and 10 (28%), respectively. Cholangiocarcinoma is the most common malignancy originating from the bile ducts and the second most common primary intrahepatic carcinoma [11]. Cholangiocarcinoma can occur in various location with heterogeneous imaging appearance on CT or MRI, and it is characterized by high biological aggressiveness and poor prognosis [11]. Recently, a growing number of radiomics applications have been proposed in patients with cholangiocarcinoma imaged with either CT or MRI, including differential diagnosis with other hepatic malignancies, prediction of lymph node metastasis, and prediction of recurrence after curative resection. However, to date the quality of radiomics studies in cholangiocarcinoma has not been comprehensively investigated. Assessment of quality in radiomics research studies is a necessary and fundamental step for the improvement of radiomics research and future implementation in clinical practice.

This systematic review aims to provide an overview of the current research applications of radiomics in patients with cholangiocarcinoma and to assess the quality of CT and MRI radiomics studies.

## Materials and methods

This study was conducted according to the Preferred Reporting Items for Systematic Reviews and Meta-analyses (PRISMA) guidelines [12]. The review protocol was registered on the International Prospective Register of Systematic Reviews (CRD42022295218).

### Literature search strategy

A systematic search was conducted to identify studies on PubMed/Medline, Web of Science, and Scopus databases using the following terms: “texture,” “radiomics,” “machine learning,” “artificial intelligence,” “cholangiocarcinoma” and “biliary cancer.” Detailed search strings are reported in the Additional file 1. The literature search was performed for articles published between 01/01/2010 and 30/11/2021.

### Eligibility criteria

After removal of duplicate studies, three Authors (R.Ca., F.V., and L.U., radiologists, each with five years of experience in radiomics studies) independently evaluated the titles and abstracts of all studies to exclude ineligible papers according to the following criteria: (1) non-English studies; (2) animal studies; (3) abstracts of conference papers; (4) reviews, systematic reviews, and case reports. The full texts of the relevant articles were read to determine their inclusion. The following eligibility criteria were applied during full-text manuscript review for the inclusion of original papers: (1) radiomics studies based on the evaluation of quantitative features obtained from tumor segmentations of cholangiocarcinoma; (2) features extracted from CT or MRI exams. Studies assessing only semantic features, other lesions than cholangiocarcinoma, or features on other diagnostic exams (i.e., ultrasound or PET/CT due to their limited applications in cholangiocarcinoma) were excluded. Any disagreement between reviewers was resolved with consensus discussion.

### Data extraction

The following data were collected from the included studies: authors, journal with its type, journal ranking according to quartile and impact factor, year of publication of the study and country based on the Institutions in which the CT/MRI of the study population exams were acquired. The journals were classified into imaging, clinical, and computer science according to the main journal category of Web of Science. The journal quartile, according to the main journal scientific sector, and impact factor were retrieved from the Journal Citation Report database, and the quartile and impact factor of the year of publication were registered. For articles published in 2021–2022, the 2020 reports were considered as this is the last available at the time of data analysis. The full manuscripts were assessed to collect the following data: type of the study (retrospective or prospective), number of involved Institutions, total number of included patients (divided into training and validation cohorts), type of cholangiocarcinoma (i.e., intrahepatic, perihilar, or extrahepatic), imaging modality (CT and/or MRI), sequences and/or phases in which the segmentation was performed, software used for segmentation and imaging analysis, and number of extracted radiomics features.

The studies were grouped according to the main purpose of investigation: diagnostic (including radiomics analysis for the differential diagnosis among hepatic lesions, prediction of tumor histopathological differentiation and markers, or lymph node involvement), prognostic (prediction of early recurrence and survival), and treatment response (response to locoregional or systemic treatments) studies.

### Radiomics quality score assessment

Three different readers from distinct Institutions and with different levels of experience (Reader 1, R1, A.P., a radiologist with 4 years of experience in radiomics research and with experience on the RQS assessment, Reader 2, R2, M.E.K., a radiologist with 10 years of experience in radiological research and 4 years of experience in radiomics research, and Reader 3, R3, E.P., a radiologist with 9 years of experience in radiological research), not involved in manuscript screening, independently evaluated all the studies using the RQS [5]. Before the manuscript assessment, a training session was held to discuss the main items of the RQS and examples on manuscripts not included in this systematic review. Both full-text manuscripts and Supplementary Materials were screened. The RQS consists of 16 items divided by three main checkpoints: the first checkpoint includes item 1, the second includes items from 2 to 4, and the third is composed by items from 5 to 16 [5]. The detailed description of the RQS is available in the Additional file 2: Table S1. The total RQS (ranging from − 8 to + 36) and the percentage of the total score (0–100%) were recorded from all three readers [5].

### Statistical analysis

Categorical variables were reported as numbers, proportions, and percentages, while continuous variables were reported as medians and interquartile ranges (IQR), after testing for normal distribution by applying the Shapiro–Wilk normality test. Adherence rate to the reporting quality of the RQS was calculated for the most experience reader (R1), considering the proportion of articles obtaining at least one point in each specific item. Differences in total RQS according to publication and study characteristics were evaluated by using the Kruskal–Wallis or the Mann–Whitney U test, as appropriate. The correlation between total RQS, journal impact factor, and number of included patients was calculated by using the Spearman’s rank correlation coefficient (Spearman’s ρ).

The intraclass correlation coefficient (ICC) with 95% confidence intervals (CI), based on an absolute-agreement with 2-way mixed-effects model, was used to assess the inter-reader agreement in the total and percentage RQS among the three readers. Agreement was categorized as poor (ICC < 0.50), moderate (ICC = 0.50–0.75), good (ICC = 0.75–0.90), or excellent (ICC > 0.90) [13].

A *p* value < 0.05 was considered to be statistically significant. Statistical analyses were conducted by using the SPSS Software (v26.0. IBM, Armonk, NY, USA).

## Results

### Literature search

**Fig. 1:**
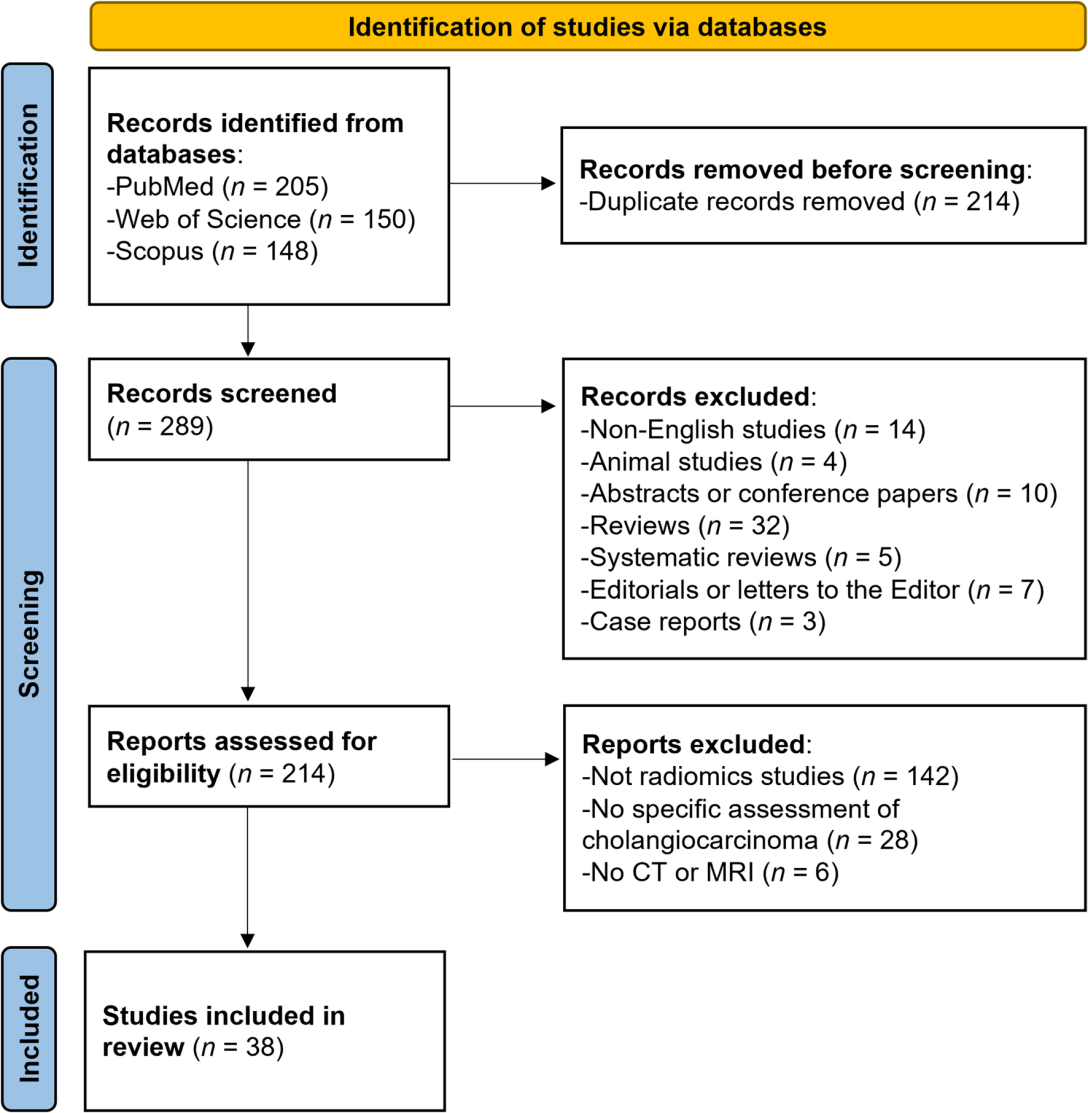
Flow diagram of the study selection process

The systematic search initially identified 503 articles (Fig. 1). After removing 214 duplicated manuscripts, 289 were screened by their title and abstracts, and 214 studies underwent full-text screening to assess their eligibility. Finally, 38 original articles on radiomics of cholangiocarcinoma were included for RQS assessment [14–51].

### Characteristics of the included studies

The characteristics of included publications are summarized in Table 1. Among the included original articles, 18/38 (47.4%) were published in an imaging journal, 15/38 (39.5%) in a clinical journal, and the remaining (5/38, 13.1%) in a computer science journal. Twenty-one (55.3%) articles were published in 2021, 9/38 (23.7%) in 2020, 5/38 (13.2%) in 2019, and 3/38 (7.8%) in 2018 or earlier years. The 17/38 (44.7%) of included articles were published in first quartile journals, with an overall median impact factor of 4.43 (IQR, 3.50–5.31). The study population most frequently originated from China (27/38, 71.1%), followed by the USA (3/38, 7.8%). Thirty (79.0%) studies were performed at one Institution, 7/38 (18.4%) were performed in two Institutions, and only one (2.6%) involved six different Centers. All the included studies were conducted retrospectively.

**Table 1:** General characteristics of the included studies

Articles	Journal	Journal type	Publication year	Quartile*	Impact factor*	Country	Centers
Chu [14]	Eur Radiol	Imaging	2021	Q1	5,315	China	Two
Duda [15]	Studies in Logic, Grammar and Rhetoric	Computer science	2013	Q2	NA	France	Two
Hamn [16]	Eur Radiol	Imaging	2019	Q1	4,101	US	Single
Huang [17]	Eur J Cancer	Clinical	2021	Q1	9,162	China	Single
Ji [18]	Eur Radiol	Imaging	2019	Q1	4,101	China	Single
Ji [19]	Radiology	Imaging	2019	Q1	7,931	China	Single
King [20]	Cancer Imaging	Imaging	2020	Q2	3,909	US	Single
Liang [21]	Front Oncol	Clinical	2018	Q2	4,137	China	Single
Liu [22]	Eur Radiol	Imaging	2021	Q1	5,315	Canada	Single
Mosconi [23]	Eur Radiol	Imaging	2020	Q1	5,315	Italy	Two
Nakai [24]	Jpn J Radiol	Imaging	2021	Q3	2,374	Japan	Single
Park [25]	Eur Radiol	Imaging	2021	Q1	5,315	Korea	Six
Park [26]	Korean J Radiol	Imaging	2021	Q2	3,500	Korea	Single
Ponnoprat [27]	Med Biol Eng Comput	Computer science	2020	Q3	2,602	Thailand	Single
Qin [28]	Liver Int	Clinical	2020	Q2	5,828	China	Two
Sadot [29]	PLoS One	Clinical	2015	Q1	3,057	US	Single
Silva [30]	Abdom Radiol	Imaging	2021	Q2	3,039	Italy	Single
Tang [31]	BMC Cancer	Clinical	2021	Q2	4,430	China	Single
Tang [32]	World J Surg Oncol	Clinical	2021	Q2	2,754	China	Single
Wang [33]	Comput Biol Med	Computer science	2021	Q1	4,598	China	Single
Wang [34]	Front Oncol	Clinical	2021	Q2	6,244	China	Two
Xu [35]	Technol Cancer Res Treat	Computer science	2021	Q3	3,399	China	Single
Xu [36]	Phys Med Biol	Imaging	2021	Q2	3,609	China	Single
Xu [37]	Theranostics	Clinical	2019	Q1	8,579	China	Single
Xue [38]	Front Oncol	Clinical	2021	Q2	6,244	China	Two
Xue [39]	Abdom Radiol	Imaging	2021	Q2	3,039	China	Two
Yang [40]	Cancer Lett	Clinical	2020	Q1	8,679	China	Single
Yao [41]	JMIR Med Inform	Computer science	2020	Q3	2,955	China	Single
Zhang [42]	ESMO Open	Clinical	2020	Q1	6,540	China	Single
Zhang [43]	Ann Transl Med	Clinical	2020	Q3	3,932	China	Single
Zhang [44]	Ann Transl Med	Clinical	2020	Q3	3,932	China	Single
Zhang [45]	Eur Radiol	Imaging	2021	Q1	5,315	China	Single
Zhao [46]	Eur J Radiol	Imaging	2021	Q2	5,315	China	Single
Zhao [47]	J Magn Reson Imaging	Imaging	2021	Q1	4,813	China	Single
Zhao [48]	Cancer Imaging	Imaging	2019	Q3	2,193	China	Single
Zhou [49]	Eur Radiol	Imaging	2021	Q1	5,315	China	Single
Zhu [50]	Sci Rep	Clinical	2021	Q1	4,380	China	Single
Zhu [51]	Sci Rep	Clinical	2021	Q2	4,380	China	Single

Journal quartile and impact factor are based on the year of publication. For articles published in 2021–2022 the 2020 data were considered

*NA* not available

**Fig. 2:**
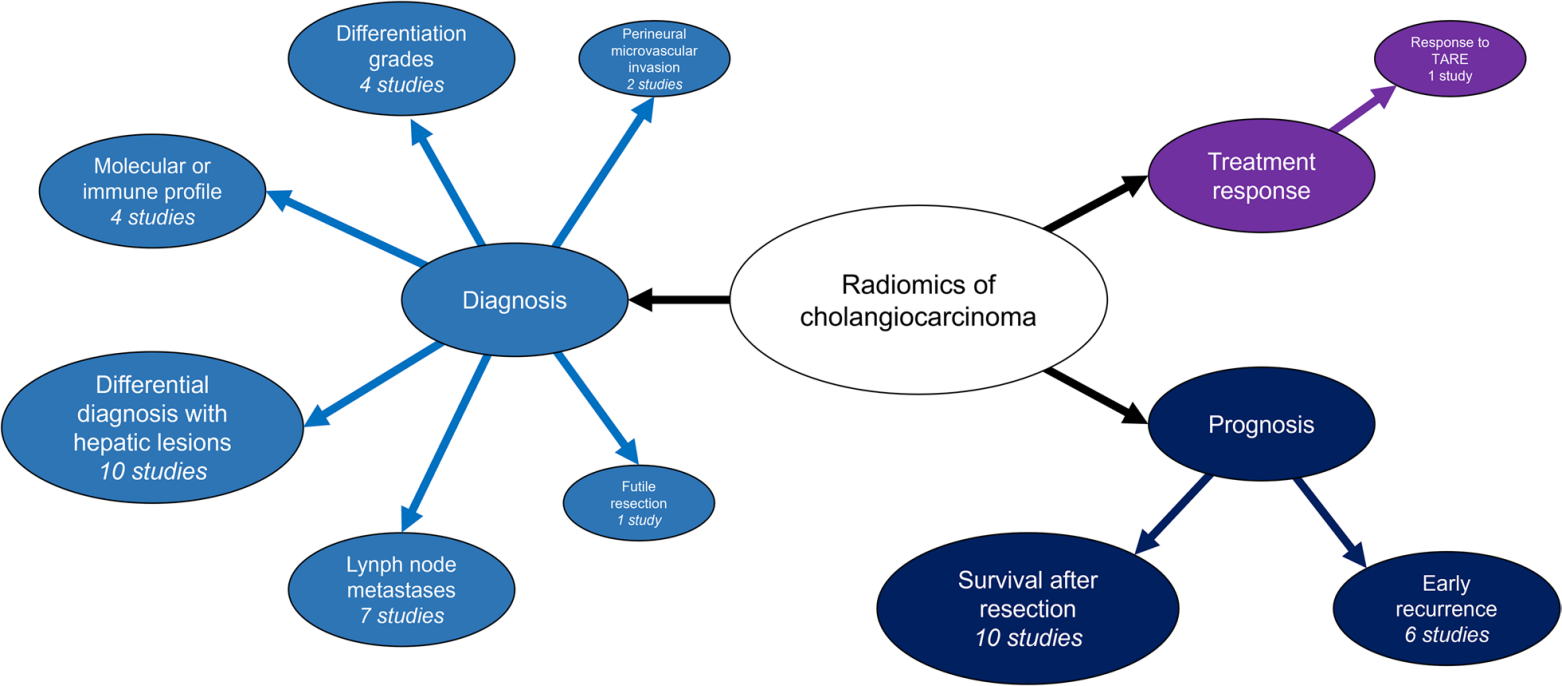
Overview of radiomics research purposes. Notably each study could include multiple purposes

Study purpose and methodology are detailed in Table 2. The most common study aims (Fig. 2) included differential diagnosis against other hepatic lesions (10/38, 26.3%), prediction of survival after surgical resection (10/38, 26.3%), and prediction of lymph node metastases (7/38, 18.4%). Only one article explored the potential of radiomics for the prediction of therapeutic response to radioembolization in intrahepatic cholangiocarcinoma [23]. The total number of patients was 6242 (median = 134 per study; IQR, 98–198). Intrahepatic, perihilar, and extrahepatic cholangiocarcinoma were assessed in 29/38 (76.3%), 4/38 (10.5%), and 5/38 (13.2%) papers, respectively. CT was the most commonly used imaging technique (20/38, 52.6%), while MRI was adopted in 16/38 (42.1%) studies. Only two (5.3%) used both techniques. Lesion segmentations for radiomics features extraction were more commonly performed in the hepatic arterial phase (27/38, 71.1%) and/or portal venous phase (25/38, 65.8%), almost always by manually drawing the region of interest (35/38, 92.1%). Segmentation of the peritumoral or adjacent hepatic parenchyma was performed in only 5/38 (13.1%) studies.

**Table 2:** Main purposes and methodology of the included studies

Articles	Purpose	CCA type	Patients (training; validation)	Imaging modality	Sequences for analysis	Segmentation method	Software	Number of features
Chu [14]	Prediction of futile resection	Intrahepatic	203 (142; 61)	CT	PVP	Manual	ITK-SNAP; A.K. software v2.0.0	1044
Duda [15]	Differential diagnosis between HCC and iCCA	Intrahepatic	76	CT	PRE-HAP-PVP	Manual	In-house	61
Hamn [16]	Differential diagnosis among hepatic lesions	Intrahepatic	494 (434; 60)	MRI	HAP-PVP-DP	Manual	Python v3.5	NA
Huang [17]	Prediction of stage, perineural, and microvascular invasion	Extrahepatic	101	MRI	T1-T2-DWI-ADC	Manual	MadZa v4.6	1208
Ji [18]	Prediction of lymph node metastasis and survival	Intrahepatic	155 (103; 52)	CT	HAP	Manual	3D Slicer v4.9.0	105
Ji [19]	Prediction of lymph node metastasis and survival	Extrahepatic	247 (177; 70)	CT	PVP	Manual	ITK-SNAP v3.6; Python	93
King [20]	Prediction of tumor grade and survival	Intrahepatic	73	CT/MRI	PRE-HAP-PVP	Manual	Osirix v5.5.2; MATLAB vR2016b	14
Liang [21]	Prediction of early recurrence	Intrahepatic	209 (139; 70)	MRI	HAP	Manual	ITK-SNAP v3.6; MATLAB R2015a	467
Liu [22]	Differential diagnosis between HCC, iCCA, and cHCC-CCA	Intrahepatic	85	CT/MRI	PRE-HAP-PVP-DP-HBP-DWI-T2-In-Phase	Manual	MintLesion + Pyradiomics v2.1.2	1419
Mosconi [23]	Prediction of response to radioembolization	Intrahepatic	55	CT	HAP-PVP-DP	Manual	LIFEx	8
Nakai [24]	Differential diagnosis between HCC and iCCA	Intrahepatic	617 (493; 62 + 62)	CT	PRE-HAP-DP	Manual	RectLabel v3.02.7; Python v3.6.4; PyTorch v1.5.0	NA
Park [25]	Prediction of survival after surgical resection	Intrahepatic	354 (233; 112)	CT	HAP-PVP	Manual	In-house (AsanFEx); MATLAB R2015a	661
Park [26]	Prediction of survival after surgical resection	Intrahepatic	89	CT	HAP	Automatic; semi-automatic	Syngo.via Frontier, RADIOMCIS prototype	19
Ponnoprat [27]	Differential diagnosis between HCC and iCCA	Intrahepatic	257	CT	PRE-HAP-PVP-DP	Automatic	NA	NA
Qin [28]	Prediction of early recurrence	Perihilar	274 (167; 70 + 37)	CT	HAP-PVP-DP	Manual	RadiAnt DICOM Viewer v4.6.5; MATLAB v9.2.0; Mazda v4.6	18,120
Sadot [29]	Correlation with molecular profile	Intrahepatic	25	CT	HAP-PVP	Semi-automatic	MATLAB	5
Silva [30]	Prediction of survival after surgical resection	Intrahepatic	78	CT	PVP	Manual	3D Slicer v4.10.2	108
Tang [31]	Prediction of lymph node metastasis and differentiation	Extrahepatic	100	MRI	T1-T2-DWI-ADC	Manual	Madza v4.6	1200
Tang [32]	Prediction of survival after surgical resection	Intrahepatic	101	CT	PVP	Manual	LIFEx v3.74	42
Wang [33]	Differential diagnosis between HCC, iCCA, and cHCC-CCA	Intrahepatic	196	MRI	HAP-PVP-DP	Manual	ITK-SNAP v3.6; Pyradiomics	1316
Wang [34]	Prediction of lymph node metastasis	Perihilar	179	CT	HAP	Manual	ITK-SNAP	1067
Xu [35]	Differential diagnosis between iCCA and lymphoma	Intrahepatic	129	CT	DP	Manual	LIFEx v3.74, Python v3.6.4	45
Xu [36]	Prediction of early and late recurrence	Intrahepatic	209 (159; 50)	MRI	T2	Manual	ITK-SNAP; MATLAB	2268
Xu [37]	Prediction of lymph node metastasis	Intrahepatic	148 (106; 42)	MRI	HAP	Manual	ITK-SNAP; MATLAB V2017b	491
Xue [38]	Diagnosis of iCCA in patients with intrahepatic lithiasis	Intrahepatic	131 (96; 35)	CT	HAP	Manual	LIFEx	52
Xue [39]	Differential diagnosis between iCCC and inflammatory masses	Intrahepatic	145 (110; 35)	CT	HAP-PVP	Manual	LIFEx	52
Yang [40]	Prediction of lymph node metastasis and differentiation	Extrahepatic	100 (80; 20)	MRI	T1-T2-DWI	Manual	MaZda v4.6	300
Yao [41]	Prediction of lymph node metastasis and differentiation	Extrahepatic	110 (88; 22)	MRI	T1-T2-DWI-ADC	Manual	MaZda v4.6	300
Zhang [42]	Prediction of PD-1/PD-L1 and survival	Intrahepatic	98	MRI	HAP-PVP	Manual	ITK-SNAP; AK software	NA
Zhang [43]	Prediction of survival after surgical resection	Intrahepatic	136	MRI	DP-DWI	Manual	AK software	384
Zhang [44]	Differential diagnosis between iCCA and cHCC-CCA	Intrahepatic	186 (132; 57)	CT	HAP-PVP	Manual	ITK-SNAP v3.6; AK software	396
Zhang [45]	Prediction of immunophenotyping and survival	Intrahepatic	78	MRI	PRE-HAP-PVP-DWI-T2	Manual	ITK-SNAP v3.6; Pyradiomics	1037
Zhao [46]	Prediction of survival after surgical resection	Perihilar	184 (110; 74)	MRI	HAP-PVP	Manual	ITK-SNAP v3.6; AK software v3.2.2	396
Zhao [47]	Prediction of early recurrence	Perihilar	184 (128; 56)	MRI	HAP-PVP	Manual	ITK-SNAP v3.6; AK software v3.2.3	402
Zhao [48]	Prediction of early recurrence	Intrahepatic	47	MRI	HAP-PVP-DP-T2	Manual	ITK-SNAP v2.2.0; AK software	396
Zhou [49]	Prediction of microvascular invasion	Intrahepatic	126 (88; 38)	MRI	HAP-PVP-DP	Manual	ITK-SNAP v3.6: Pyradiomics v2.12	2364
Zhu [50]	Prediction of IDH mutation	Intrahepatic	138	CT	PRE-HAP-PVP-DP	Manual	Pyradiomics	72
Zhu [51]	Prediction of early recurrence	Intrahepatic	125 (92; 33)	CT	PRE-HAP-PVP-DP	Manual	Pyradiomics	87

*ADC* apparent diffusion coefficient; *CCA* cholangiocarcinoma; *cHCC-CCA* combined hepatocellular-cholangiocarcinoma; *CT* computed tomography; *DP* delayed phase; *DWI* diffusion weighted imaging; *HAP* hepatic arterial phase; *HBP* hepatobiliary phase; *HCC* hepatocellular carcinoma; *iCCA* intrahepatic cholangiocarcinoma; *MRI* magnetic resonance imaging; *NA* not available; *PRE* pre-contrast phase; *PVP* portal-venous phase

### Radiomics quality score

Results of the total RQS by the three independent readers are summarized in Table 3. Details on the items’ score by each reader are provided in the Additional file 2: Tables S2, S3, and S4. The median RQS was 9 (corresponding to the 25.0% of the total RQS; IQR 1–13) for R1, 8 (22.2%, IQR 3–12) for R2, and 10 (27.8%; IQR 5–14) for R3. The inter-reader agreement for was good with an ICC of 0.75 (95% CI 0.62–0.85) for the total RQS and 0.77 (95% CI 0.65–0.86) for the RQS percentage scores.

**Table 3:** Total radiomics quality score (RQS) with percentage of the total score of the included studies assessed by three independent readers

	**Reader 1**		**Reader 2**		**Rader 3**	
**Articles**	**Total**	**Percentage**	**Total**	**Percentage**	**Total**	**Percentage**
Chu [14]	11	30.6%	11	30.6%	14	38.9%
Duda [15]	1	2.8%	−1	0%	−6	0%
Hamn [16]	3	8.3%	8	22.2%	7	19.4%
Huang [17]	8	22.2%	4	11.1%	5	13.9%
Ji [18]	16	44.4%	13	36.1%	15	41.7%
Ji [19]	14	38.9%	15	41.7%	14	38.9%
King [20]	−4	0%	−5	0%	2	5.6%
Liang [21]	12	33.3%	14	38.9%	6	16.7%
Liu [22]	1	2.8%	5	13.9%	−2	0%
Mosconi [23]	0	0%	5	13.9%	−2	0%
Nakai [24]	4	11.1%	6	16.7%	14	38.9%
Park [25]	18	50.0%	18	50.0%	17	47.2%
Park [26]	−2	0%	3	8.3%	−4	0%
Ponnoprat [27]	9	25.0%	2	5.6%	11	30.6%
Qin [28]	15	41.7%	11	30.6%	15	41.7%
Sadot [29]	−5	0%	−4	0%	−1	0%
Silva [30]	−1	0%	5	13.9%	−3	0%
Tang [31]	8	22.2%	7	19.4%	13	36.1%
Tang [32]	13	36.1%	8	22.2%	15	41.7%
Wang [33]	−1	0%	−1	0%	2	5.6%
Wang [34]	12	33.3%	14	38.9%	13	36.1%
Xu [35]	9	25.0%	10	27.8%	12	33.3%
Xu [36]	10	27.8%	9	25.0%	7	19.4%
Xu [37]	16	44.4%	13	36.1%	13	36.1%
Xue [38]	13	36.1%	15	41.7%	13	36.1%
Xue [39]	11	30.6%	16	44.4%	14	38.9%
Yang [40]	8	22.2%	7	19.4%	9	25.0%
Yao [41]	9	25.0%	9	25.0%	11	30.6%
Zhang [42]	8	22.2%	2	5.6%	5	13.9%
Zhang [43]	2	5.6%	6	16.7%	4	11.1%
Zhang [44]	15	41.7%	12	33.3%	14	38.9%
Zhang [45]	3	8.3%	−1	0%	9	25.0%
Zhao [46]	15	41.7%	12	33.3%	6	16.7%
Zhao [47]	15	41.7%	13	36.1%	15	41.7%
Zhao [48]	−2	0%	−2	0%	6	16.7%
Zhou [49]	9	25.0%	11	30.6%	12	33.3%
Zhu [50]	−5	0%	3	8.3%	11	30.6%
Zhu [51]	13	36.1%	12	33.3%	9	25.0%
Median (IQR)	9 (1–13)	25.0 (2.8–36.1)	8 (3–12)	22.1 (8.3–33.3)	10 (5–14)	27.8 (13.9–38.9)

**Fig. 3:**
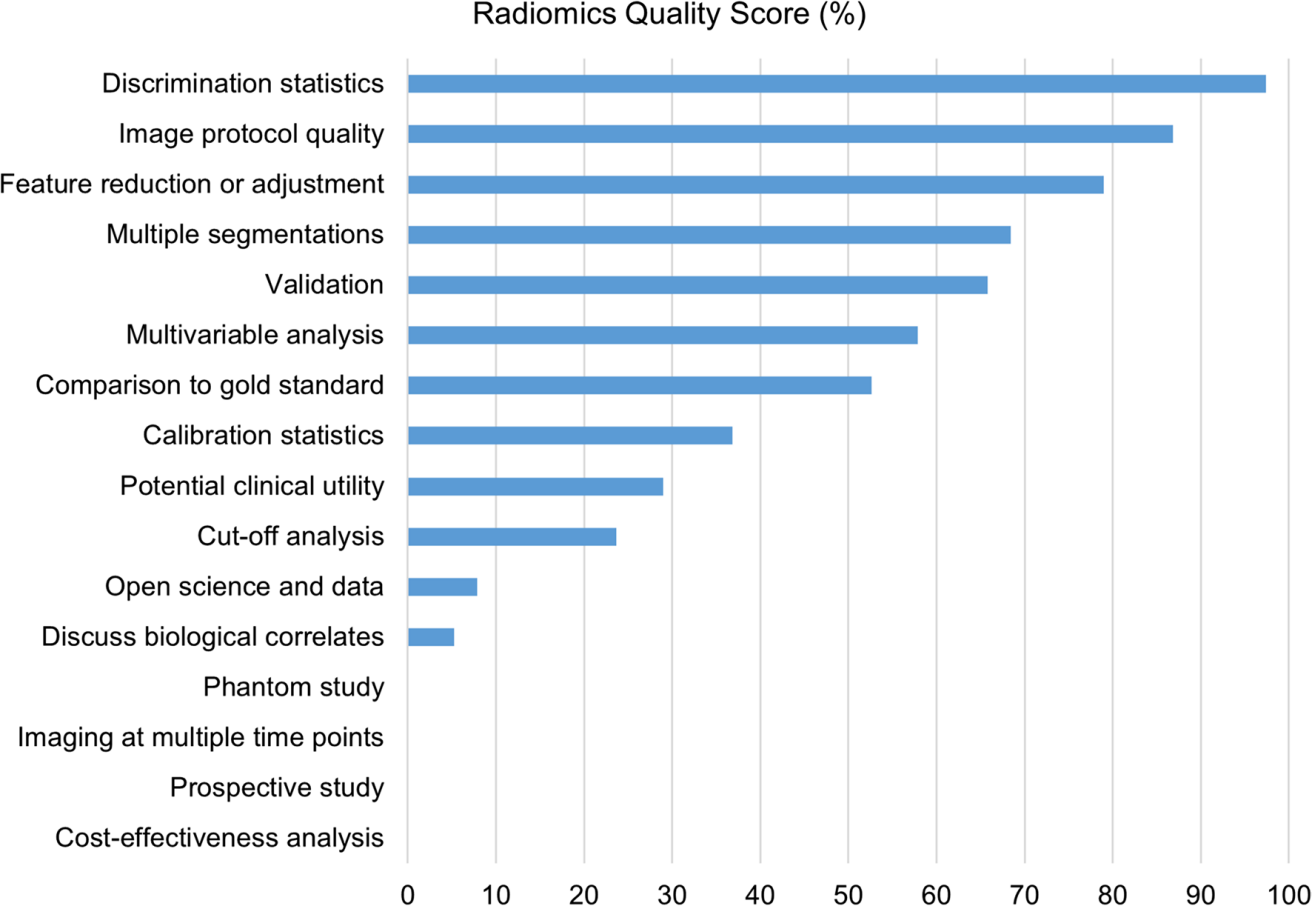
Adherence rate to the reporting quality of each item included in the radiomics quality score according to the most experienced reader (R1)

Adherence rate of each item (according to R1) is illustrated in Fig. 3. For the first checkpoint (item 1), 33/38 (88.6%) studies provided a well-documented image protocol. In the second checkpoint (items from 2 to 4), 26/38 (68.4%) studies had multiple segmentations, but none performed phantom assessment or imaging at multiple time points. In the third checkpoint (items from 5 to 16), feature reduction and adjustment for multiple tests was employed in 30/38 (78.9%) cases, with non-radiomics features included in 22/38 (57.9%) multivariate analyses. Only two (5.3%) articles discussed biological correlates related to the radiomics models. Cutoff analyses, discrimination statistics, and calibration statistics were available in 9/38 (23.7%), 37/38 (97.4%), and 14/38 (36.8%) investigations, respectively. Regarding the validation of the radiomics models, in most studies (23/38, 60.5%) it was based on an internal cohort, but validation was lacking in 13/38 (34.2%) investigations. Comparison with the gold standard and discussion of potential clinical utility were addressed in 20/38 (52.6%) and 11/38 (28.9%) studies, respectively. None of the assessed article was prospectively registered in a trial database or provided cost-effectiveness analysis, and only three (7.9%) made their code or data publicity available.

### Subgroup analyses

Results of the subgroup analyses are reported in Table 4. No statistical differences were found according to the type of journal, year of publication, journal quartile, type of included cholangiocarcinoma, and imaging modalities. None of the RQS items (according to R1) was significantly higher in first quartile journals (*p* ≥ 0.101). Journals with impact factors > 4 published studies with significantly higher RQS according to the R1 (*p* = 0.048) and R2 (*p* = 0.035). The RQS was significantly higher in studies including more than 100 patients (*p* < 0.001 for all the readers).

**Table 4:** Subgroup analyses of total radiomics quality score assessed by the three independent readers

		Reader 1		Reader 2		Reader 3	
Group	N	RQS total	*p* value	RQS total	*p* value	RQS total	*p* value
Journal type			0.542		0.302		0.562
Imaging	18	6.5 (0–14)		8.5 (4.5–13)		8 (1–14)	
Clinical	15	12 (8–13)		8 (4–13)		11 (5–13)	
Computer science	5	9 (1–9)		2 (−1 to 9.5)		11 (−2 to 11.5)	
Publication year			0.772		0.322		0.352
2021	21	9 (2–13)		9 (4.5–12.5)		12 (5.5–14)	
2013–2020	17	8 (0.5–14.5)		7 (0.5–12.5)		7 (3–13.5)	
Journal quartile			0.787		0.510		0.814
Q1	17	8 (0.5–14.5)		7 (2.5–13)		9 (3.5–14)	
Q2	14	11.5 (0.5–13)		10 (4.5–14)		8 (0.7–13.2)	
Q3	7	9 (2–9)		6 (2–10)		11 (6–14)	
Impact factor			**0.048**		**0.035**		0.224
≤ 4	15	4 (−2 to 10)		6 (−1 to 9)		7 (−1 to 14)	
> 4	23	11 (3–15)		11 (5–13)		11 (6–14)	
Cholangiocarcinoma			0.074		0.124		0.152
Intrahepatic	29	8 (−0.5 to 12.5)		6 (2–12)		9 (2–13.5)	
Perihilar/extrahepatic	9	12 (8–15)		11 (7–13.5)		13 (7.5–14.5)	
Imaging modality			0.361		0.206		0.067
CT	20	11 (0.2–13.7)		10.5 (3.5–13.7)		13 (1.5–14)	
MRI/MRI and CT	18	8 (1.7–10.5)		7 (1.2–11.2)		6.5 (4.7–11.2)	
Number of patients			** < 0.001**		** < 0.001**		** < 0.001**
≤ 100	12	0.5 (−2 to 6.7)		2.5 (−1.7 to 5)		0.5 (−2.7 to 8.2)	
> 100	26	11.5 (8.7–15)		11 (7.5–13.2)		12.5 (7–14)	

Continuous variables are expressed as medians and interquartile range (25th to 75th  percentile) in parenthesis. Continuous variables were compared using the Kruskal–Wallis or the Mann–Whitney U test. Statistically significant values (*p* < 0.05) are highlighted in bold

CT, Computed Tomography; MRI, Magnetic Resonance Imaging

For all the three readers, there was a statistically significant high correlation between the total RQS and number of included patients (*p* < 0.001 for all the readers) (**Table ****5**). No significant correlation was observed between the total RQS and other characteristics.

**Table 5:** Correlation between total radiomics quality score assessed by three independent readers, journal impact factor, number of included patients, and number or radiomics features

	**Reader 1**	**Reader 2**	**Reader 3**
Journal impact factor *p* value	0.315 0.057	0.288 0.083	0.089 0.602
Number of patients *p* value	0.593 ** < 0.001**	0.587 ** < 0.001**	0.596 ** < 0.001**
Number of features *p* value	0.265 0.130	0.160 0.368	0.200 0.256

Numbers represent the Spearman’s rank correlation coefficient (ρ), unless otherwise specified. Statistically significant values (*p* < 0.05) are highlighted in bold

## Discussion

Inadequate quality of radiomics studies is emerging as a major issue of current literature, contributing to the slow transition from research to clinical application in this field [52]. This systematic review of 38 radiomics studies on cholangiocarcinoma demonstrates a suboptimal quality of the current publications assessed through the RQS, with an overall total score of 8–10, corresponding to about one quarter of the ideal quality for this type of study. This is in line with other systematic reviews based on the RQS assessing radiomics of hepatic lesions, reporting a median RQS of 8–14 corresponding to 23–39% of the total score [6–10]. Importantly, in this review none of the included studies had phantom assessment, imaging at multiple time points, prospective registration in a trial database, nor performed cost-effectiveness analysis. These items account for 10 points (28%) of the total RQS [5].

Radiomics has been applied as a diagnostic tool for the differential diagnosis between cholangiocarcinoma and other hepatic tumors, for preoperative identification of histopathological and molecular markers associated with poor prognosis, and for predicting postoperative survival, while there is still a very limited experience on therapeutic response and advanced lesions that were not suitable for surgical resection [23]. To date, all studies on radiomics of cholangiocarcinoma are retrospective, mostly based on a single-center dataset with lack of validation cohorts in 34% of them. This is a relevant issue, determining a loss of 5 points in the total RQS, as external validation is a key item prior to clinical implementation of classification models. Only the study by Park et al. [25] validated a radiomics model for the prediction of postoperative outcome in patients with intrahepatic cholangiocarcinoma in an external test dataset from five different institutions (obtaining the maximum score of + 5 points in item 12 of the RQS). This means that even though most studies focused on radiomics of cholangiocarcinoma have a great potential, their results are still confined to the academic centers where the model originated. Further investigations should focus on the validation of existing models in a multicentric context rather than proposing alternative models based on a single-center experiences. Prospective validation of the radiomics models is also needed to evaluate their potential in clinical practice focusing on relevant patients’ outcomes such us evaluation of overall survival after treatment. In this setting, open science data providing the code and radiomics data is of utmost importance to facilitate the widespread application of radiomics and the reproducibility of the proposed models. Nevertheless, less than 10% of studies included in this systematic review made their code or data publicity available.

Despite the need of well-conducted radiomics workflow has been emphasized over the last years, the quality of published radiomics papers on cholangiocarcinoma according to the RQS has not increased when comparing 2021 versus 2013–2020. High-impact journals have provided guidance highlighting the need for robust data and accurate methodology for radiomics research [1, 53, 54]. However, the analysis of current cholangiocarcinoma studies demonstrates no significant difference in the RQS based on the journal type or quartile, even though a tendency of higher RQS was observed in journals with impact factor greater than 4 in two out of three readers. In prior studies, no difference according to journal metrics were found by Spadarella et al. [55] in RQS of nasopharyngeal cancer studies, while Ponsiglione et al. [56] and Chang et al. [57] observed significantly higher RQS in journals with higher impact factor or quartile for cardiac imaging studies, respectively. Therefore, the explosion of research on radiomics, machine learning, and data-based science led to an increased number of published radiomics papers not followed by a significant increase in quality of those studies. This may be related to the overall tendency to perform and publish explorative radiomics studies based on the novelty of the topic rather than to improve the strict methodology and workflow of radiomics analysis [52, 58]. Standardization of the acquisition protocols in liver imaging is a fundamental effort in order to minimize the variability of radiomics features across centers and scanners [59]. Furthermore, the International Biomarker Standardization Initiative (IBSI) is working toward standardization of extraction of quantitative features extracted from medical images and it already provided reference values for radiomics features on CT [60].

The RQS provides a detailed description of each item’s score [5]. However, its application can be affected by the reader’s experience and interpretation of each item according to the data available in the papers. All the studies included in this review were evaluated by three independent readers with different levels of research experience and RQS assessment, which resulted in a good inter-observer agreement. Few studies evaluated the reproducibility of the RQS with discordant results (reported ICC between 0.57 and 0.99) and, to our knowledge, none of these studies evaluated the inter-observed agreement in readers from different Institutions [55, 61–63]. It should be noted that the RQS is based on expert opinion and currently not endorsed by scientific societies and it is limited by strong dependence on the methodological quality of the ideal radiomics workflow with low relevance to the potential clinical impact. Some of the items, such as phantom assessment and imaging at multiple time points, remain difficult to be investigated when considering real-word data based on retrospective observational studies. Nevertheless, the application of this score could be encouraged for the quality assessment of the papers submitted to peer-review journals in order to facilitate manuscript decisions and improve the overall quality of radiomics studies.

Some limitations pertain to this study. First, a meta-analysis was not performed due to the heterogeneity of the included studies, with a relatively small number of papers assessing the radiomics models for a specific aim, which makes challenging to pool data for a strong meta-analysis. Secondly, cholangiocarcinomas are rarer tumors compared to hepatocellular carcinoma and hepatic metastasis, and the applications of the radiomics in this field is relatively new. This is demonstrated by the fact that 55% of the included studies were published in 2021. Finally, papers with radiomics applied to cholangiocarcinoma on ultrasound and PET/CT were not included due to the limited clinical applicability in patients with cholangiocarcinoma and highly exploratory nature of radiomics analyses with these imaging modalities.

In conclusion, radiomics studies on cholangiocarcinoma demonstrated an insufficient quality with a low total radiomics quality score. Further prospective studies are needed with a standardized methodology, validation in multi-imitational external cohorts, and open science data in order to translate the promising research results in the field of radiomics into useful applications to improved patients’ management in many clinical scenarios.

## Supplementary Information

**Additional file 1.** Detailed search strategy.

**Additional file 2. Table S1:** Detailed checklist of the radiomics quality score with corresponding checkpoints and items as reported by Lambin et al [5]. **Table S2:** Radiomics quality score of the included studies assessed by the Reader 1. **Table S3:** Radiomics quality score of the included studies assessed by the Reader 2. **Table S4:** Radiomics quality score of the included studies assessed by the Reader 3.

## Data Availability

The datasets used and/or analyzed during the current study are available from the corresponding author on reasonable request.

## Abbreviations

CI: Confidence interval
CT: Computed tomography
IBSI: International Biomarker Standardization Initiative
ICC: Intraclass correlation coefficient
IQR: Interquartile range
MRI: Magnetic resonance imaging
RQS: Radiomics quality score
