# Supplementary material for: Systematic review with radiomics quality score of cholangiocarcinoma: an EuSoMII Radiomics Auditing Group Initiative
Source: Insights Imaging. 2023 Feb 1;14:21. doi: 10.1186/s13244-023-01365-1 (PMC9889586; doi:10.1186/s13244-023-01365-1)
Supplement: Supplementary file 1 — Additional file 1. Detailed search strategy. [file 13244_2023_1365_MOESM1_ESM.docx]

# SUPPLEMENTARY MATERIAL

***Detailed search strategy***

**PubMed**: (((((texture) OR (radiomics)) OR (machine learning)) OR (artificial intelligence)) AND ((cholangiocarcinoma) OR (biliary cancer))) AND (("2010/01/01"[Date - Publication] : "2021/11/30"[Date - Publication]))

**Web of Science**: (((ALL=(texture)) OR ALL=(radiomics)) OR ALL=(machine learning)) OR ALL=(artificial intelligence) AND (ALL=(cholangiocarcinoma)) OR ALL=(biliary cancer) AND DOP=(2010-01-01/2021-11-30)

**Scopus**: ( TITLE-ABS-KEY ( texture ) OR TITLE-ABS-KEY ( radiomics ) OR TITLE-ABS-KEY ( machine AND learning ) OR TITLE-ABS-KEY ( artificial AND intelligence ) AND TITLE-ABS-KEY ( cholangiocarcinoma ) OR TITLE-ABS-KEY ( biliary AND cancer ) ) AND PUBYEAR > 2009
