# Supplementary material for: Systematic review with radiomics quality score of cholangiocarcinoma: an EuSoMII Radiomics Auditing Group Initiative
Source: Insights Imaging. 2023 Feb 1;14:21. doi: 10.1186/s13244-023-01365-1 (PMC9889586; doi:10.1186/s13244-023-01365-1)
Supplement: Supplementary file 2 — Additional file 2. Table S1: Detailed checklist of the radiomics quality score with corresponding checkpoints and items as reported by Lambin et al [5]. Table S2: Radiomics quality score of the included studies assessed by the Reader 1. Table S3: Radiomics quality score of the included studies assessed by the Reader 2. Table S4: Radiomics quality score of the included studies assessed by the Reader 3. [file 13244_2023_1365_MOESM2_ESM.pdf]

ELECTRONIC SUPPLEMENTARY MATERIAL

Systematic review with radiomics quality score of cholangiocarcinoma: an EuSoMII Radiomics Auditing Group Initiative

Supplementary Table 1: Detailed checklist of the radiomics quality score with corresponding checkpoints and items as reported by Lambin et al [5].

Checkpoints	Items	Scores
Checkpoint 1	Item 1: Image protocol quality	+1 if protocols are well-documented; +1 if public protocol is used.
Checkpoint 2	Item 2: Multiple segmentations	+1 if segmentation by different physicians/algorithms/software, perturbing segmentations by (random) noise, segmentation at different breathing cycles.
	Item 3: Phantom study	+1 if detect inter-scanner differences and vendor-dependent features.
	Item 4: Imaging at multiple time points	+1 if analyze feature robustness to temporal variabilities (for example, organ movement, organ expansion/shrinkage).
Checkpoint 3	Item 5: Feature reduction or adjustment for multiple testing	-3 if neither measure is implemented; +3 if either measure is implemented.
	Item 6: Multivariable analysis with non-radiomics features	+1 if non-radiomics features are included in the model.
	Item 7: Detect and discuss biological correlates	+1 if discuss biological correlates.
	Item 8: Cut-off analyses	+1 if determine risk groups by either the median, a<br>previously published cut-off or report a continuous risk variable.
	Item 9: Discrimination statistics	+1 if a discrimination statistic and its statistical significance are reported; +1 if a resampling method technique is also applied.
	Item 10: Calibration statistics	+1 if a calibration statistic and its statistical significance are reported; +1 if a resampling method technique is also applied.

	Item 11: Prospective study registered in a trial database	+7 if prospective validation of a radiomics signature in an appropriate trial.
	Item 12: Validation	-5 if validation is missing; +2 if validation is based on a dataset from the same institute; + 3 if validation is based on a dataset from another institute; + 4 if validation is based on two datasets from two distinct institutes; + 4 if the study validates a previously published signature; + 5 if validation is based on three or more datasets from distinct institutes
	Item 13: Comparison to gold standard	+2 if assess the extent to which the model agrees with/is superior to the current 'gold standard' method.
	Item 14: Potential clinical utility	+2 if report on the current and potential application of the model in a clinical setting.
	Item 15: Cost-effectiveness analysis	+1 if report on the cost-effectiveness of the clinical application.
	Item 16: Open science and data	+1 if scans are open source; +1 if region of interest segmentations are open source; +1 if code is open source; +1 if radiomics features are calculated on a set of representative ROIs and the calculated features and representative ROIs are open source.

Supplementary Table 2: Radiomics quality score of the included studies assessed by the Reader 1.

	Item 1	Item 2	Item 3	Item 4	Item 5	Item 6	Item 7	Item 8	Item 9	Item 10	Item 11	Item 12	Item 13	Item 14	Item 15	Item 16	RQS (%)
Chu [14]	1	1	0	0	3	1	0	0	2	0	0	2	2	0	0	0	11 (30.6%)
Duda [15]	1	1	0	0	3	0	0	0	0	0	0	-5	0	0	0	0	1 (2.8%)
Hamn [16]	1	0	0	0	-3	0	0	0	2	0	0	2	2	0	0	0	3 (8.3%)
Huang [17]	1	1	0	0	3	0	0	0	0	0	0	2	0	0	0	0	8 (22.2%)
Ji [18]	1	1	0	0	3	1	0	1	2	2	0	2	2	2	0	0	16 (44.4%)
Ji [19]	1	0	0	0	3	0	0	1	2	2	0	2	2	2	0	0	14 (38.9%)
King [20]	0	0	0	0	-3	1	0	0	2	0	0	-5	2	0	0	0	-4 (0%)
Liang [21]	1	1	0	0	3	1	0	0	0	2	0	2	0	2	0	0	12 (33.3%)
Liu [22]	1	0	0	0	3	0	0	0	0	0	0	-5	0	0	0	0	1 (2.8%)
Mosconi [23]	0	0	0	0	3	1	0	0	0	0	0	-5	0	0	0	0	0 (0%)
Nakai [24]	1	0	0	0	-3	0	0	0	2	0	0	2	2	0	0	0	4 (11.1%)
Park [25]	1	1	0	0	3	1	0	0	2	2	0	5	2	2	0	0	18 (50.0%)
Park [26]	1	1	0	0	-3	1	0	0	2	0	0	-5	2	0	0	0	-2 (0%)
Ponnoprat[27]	2	0	0	0	3	0	0	0	0	0	0	2	0	0	0	1	9 (25.0%)
Qin [28]	0	1	0	0	3	1	0	0	2	0	0	4	2	0	0	1	15 (41.7%)
Sadot [29]	1	0	0	0	-3	0	1	0	0	0	0	-5	0	0	0	0	-5 (0%)
Silva [30]	0	1	0	0	-3	1	0	1	2	0	0	-5	2	0	0	0	-1 (0%)
Tang [31]	1	0	0	0	3	0	0	0	0	0	0	2	0	0	0	1	8 (22.2%)
Tang [32]	1	1	0	0	3	1	0	1	2	0	0	2	2	0	0	0	13 (36.1%
Wang [33]	0	0	0	0	3	0	0	0	0	0	0	-5	0	0	0	0	-1 (0%)

Wang [34]	1	1	0	0	3	1	0	0	2	0	0	2	2	0	0	0	12 (33.3%)
Xu [35]	1	1	0	0	3	0	0	0	0	0	0	2	0	0	0	0	9 (25.0%)
Xu [36]	1	0	0	0	3	1	0	0	2	0	0	2	2	0	0	0	10 (27.8%)
Xu [37]	1	1	0	0	3	1	0	1	2	2	0	2	2	2	0	0	16 (44.4%)
Xue [38]	1	1	0	0	3	1	0	0	0	2	0	2	0	2	0	0	13 (36.1%)
Xue [39]	1	1	0	0	3	1	0	0	0	0	0	2	0	0	0	0	11 (30.6%)
Yang [40]	1	1	0	0	3	0	0	0	0	0	0	2	0	0	0	0	8 (22.2%)
Yao [41]	1	1	0	0	3	0	0	0	0	0	0	2	0	0	0	0	9 (25.0%)
Zhang [42]	1	1	0	0	3	1	1	1	2	0	0	-5	2	0	0	0	8 (22.2%)
Zhang [43]	1	0	0	0	3	1	0	0	0	2	0	-5	0	2	0	0	2 (5.6%)
Zhang [44]	1	1	0	0	3	1	0	0	2	2	0	2	2	2	0	0	15 (41.7%)
Zhang [45]	1	1	0	0	3	0	0	1	0	0	0	-5	0	0	0	0	3 (8.3%)
Zhao [46]	1	1	0	0	3	1	0	0	2	2	0	2	2	2	0	0	15 (41.7%)
Zhao [47]	1	1	0	0	3	1	0	1	2	2	0	2	2	2	0	0	15 (41.7%)
Zhao [48]	1	1	0	0	-3	1	0	0	2	0	0	-5	2	0	0	0	-2 (0%)
Zhou [49]	1	1	0	0	3	0	0	1	0	0	0	2	0	0	0	0	9 (25.0%)
Zhu [50]	1	1	0	0	-3	0	0	0	0	0	0	-5	0	0	0	0	-5 (0%)
Zhu [51]	1	1	0	0	3	1	0	0	2	2	0	2	2	2	0	0	13 (36.1%)

Supplementary Table 3: Radiomics quality score of the included studies assessed by the Reader 2.

	Item 1	Item 2	Item 3	Item 4	Item 5	Item 6	Item 7	Item 8	Item 9	Item 10	Item 11	Item 12	Item 13	Item 14	Item 15	Item 16	RQS (%)
Chu [14]	1	1	0	0	3	1	0	0	1	0	0	2	0	2	0	0	11 (30.6%)
Duda [15]	0	0	0	0	3	0	0	0	1	0	0	-5	0	0	0	0	-1 (0%)
Hamn [16]	1	0	0	1	0	0	1	0	1	0	0	2	2	0	0	0	8 (22.2%)
Huang [17]	1	1	0	0	3	0	1	0	1	0	0	-5	0	2	0	0	4 (11.1%9
Ji [18]	1	1	0	0	3	1	1	0	2	0	0	2	0	2	0	0	13 (36.1%)
Ji [19]	1	1	0	0	3	0	1	0	2	1	0	2	2	2	0	0	15 (41.7%)
King [20]	0	1	0	0	-3	0	1	0	1	0	0	-5	0	0	0	0	-5 (0%)
Liang [21]	1	1	0	0	3	1	1	0	2	1	0	2	0	2	0	0	14 (38.9%)
Liu [22]	1	0	0	0	3	0	0	0	2	0	0	-5	2	2	0	0	5 (13.9%)
Mosconi [23]	0	1	1	0	3	0	1	0	2	0	0	-5	0	2	0	0	5 (13.9%)
Nakai [24]	1	0	0	0	-3	1	1	0	2	0	0	2	2	0	0	0	6 (16.7%)
Park [25]	1	1	0	0	3	0	1	0	2	1	0	5	2	2	0	0	18 (50.0%)
Park [26]	1	1	0	0	3	1	0	0	0	0	0	-5	0	2	0	0	3 (8.3%)
Ponnoprat[27]	1	0	0	0	-3	0	0	0	0	0	0	2	0	2	0	0	2 (5.6%)
Qin [28]	0	1	0	0	-3	1	0	1	2	1	0	3	2	2	0	1	11 (30.6%)
Sadot [29]	1	0	0	0	-3	0	1	0	0	0	0	-5	0	2	0	0	-4 (0%)
Silva [30]	0	1	0	0	3	0	0	1	2	1	0	-5	0	2	0	0	5 (13.9%)
Tang [31]	1	0	0	0	3	0	0	0	1	0	0	2	0	0	0	0	7 (19.4%)

Tang [32]	1	0	0	0	3	0	0	0	0	0	0	2	0	2	0	0	8 (22.2%9
Wang [33]	0	0	0	0	3	0	0	0	1	0	0	-5	0	0	0	0	-1 (0%)
Wang [34]	1	0	0	0	3	1	0	0	2	2	0	3	0	2	0	0	14 (38.9%)
Xu [35]	1	1	0	0	3	0	1	0	2	0	0	2	0	0	0	0	10 (27.8%)
Xu [36]	0	1	0	0	3	1	0	1	1	0	0	2	0	0	0	0	9 (25.0%)
Xu [37]	1	1	0	0	3	1	0	0	2	1	0	2	0	2	0	0	13 (36.1%)
Xue [38]	1	1	0	0	3	1	0	0	1	2	0	3	0	2	0	1	15 (41.7%)
Xue [39]	1	1	0	0	3	1	0	0	2	2	0	3	0	2	0	1	16 (44.4%)
Yang [40]	1	1	0	0	3	0	0	0	0	0	0	2	0	0	0	0	7 (19.4%)
Yao [41]	1	1	0	0	3	0	0	0	2	0	0	2	0	0	0	0	9 (25.0%)
Zhang [42]	0	0	0	0	3	1	1	0	2	0	0	-5	0	0	0	0	2 (5.6%)
Zhang [43]	1	0	0	0	3	1	1	0	1	2	0	-5	0	2	0	0	6 (16.7/%)
Zhang [44]	0	1	0	0	3	1	0	0	1	2	0	2	0	2	0	0	12 (33.3%)
Zhang [45]	1	1	0	0	-3	0	1	0	1	1	0	-5	0	2	0	0	-1 (0%)
Zhao [46]	1	1	0	0	3	0	0	0	1	2	0	2	0	2	0	0	12 (33.3%)
Zhao [47]	1	1	0	0	3	1	0	0	1	2	0	2	0	2	0	0	13 (36.1%)
Zhao [48]	1	1	0	0	-3	1	0	0	1	0	0	-5	0	2	0	0	-2 (0%)
Zhou [49]	1	1	0	0	3	0	1	0	1	2	0	2	0	0	0	0	11 (30.6%)
Zhu [50]	0	1	0	0	-3	0	1	0	2	0	0	2	0	0	0	0	3 (8.3%)
Zhu [51]	0	1	0	0	3	1	1	0	2	0	0	2	0	2	0	0	12 (33.3%)

Supplementary Table 4: Radiomics quality score of the included studies assessed by the Reader 3.

	Item 1	Item 2	Item 3	Item 4	Item 5	Item 6	Item 7	Item 8	Item 9	Item 10	Item 11	Item 12	Item 13	Item 14	Item 15	Item 16	RQS (%)
Chu [14]	1	1	0	0	3	1	0	1	2	0	0	3	0	2	0	0	14 (38.9%)
Duda [15]	1	0	0	0	-3	0	0	0	1	0	0	-5	0	0	0	0	-6 (0%)
Hamn [16]	1	1	0	0	3	0	0	0	0	0	0	2	0	0	0	0	7 (19.4%)
Huang [17]	0	1	0	0	3	0	0	0	2	0	0	-5	2	2	0	0	5 (13.9%)
Ji [18]	1	1	0	0	3	1	0	1	2	2	0	2	0	2	0	0	15 (41.7%)
Ji [19]	1	1	0	0	3	0	0	1	2	2	0	2	0	2	0	0	14 (38.9%)
King [20]	0	1	0	0	-3	0	0	0	2	0	0	2	0	0	0	0	2 (5.6%)
Liang [21]	1	1	0	0	3	0	0	0	2	2	0	-5	0	2	0	0	6 (16.7%)
Liu [22]	1	1	0	0	-3	0	0	0	2	0	0	-5	0	2	0	0	-2 (0%)
Mosconi [23]	1	1	0	0	-3	0	0	1	1	0	0	-5	0	2	0	0	-2 (0%)
Nakai [24]	1	1	0	0	3	1	0	0	2	2	0	2	0	2	0	0	14 (38.9%)
Park [25]	1	1	0	0	3	0	0	1	2	2	0	5	0	2	0	0	17 (47.2%)
Park [26]	1	1	0	0	-3	0	0	0	2	0	0	-5	0	0	0	0	-4 (0%)
Ponnoprat[27]	1	1	0	0	3	0	0	0	2	0	0	2	0	2	0	0	11 (30.6%)
Qin [28]	0	1	0	0	3	1	0	1	2	2	0	3	0	2	0	0	15 (41.7%)
Sadot [29]	1	0	0	0	-3	1	1	0	2	0	0	-5	0	2	0	0	-1 (0%)
Silva [30]	0	1	0	0	-3	0	0	1	1	0	0	-5	0	2	0	0	-3 (0%)
Tang [31]	1	0	0	0	3	1	0	1	2	0	0	2	0	2	0	1	13 (36.1%)
Tang [32]	1	1	0	0	3	1	0	1	2	2	0	2	0	2	0	0	15 (41.7%)

Wang [33]	0	0	0	0	3	1	0	0	1	0	0	-5	0	2	0	0	2 (5.6%)
Wang [34]	1	1	0	0	3	1	0	0	1	0	0	3	1	2	0	0	13 (36.1%)
Xu [35]	1	1	0	0	3	1	0	0	2	0	0	2	0	2	0	0	12 (33.3%)
Xu [36]	0	1	0	0	3	0	0	0	1	0	0	2	0	0	0	0	7 (19.4%)
Xu [37]	1	1	0	0	3	0	0	0	2	2	0	2	0	2	0	0	13 (36.1%)
Xue [38]	1	1	0	0	3	0	0	0	2	2	0	2	0	2	0	0	13 (36.1%)
Xue [39]	1	1	0	0	3	1	0	0	2	2	0	2	0	2	0	0	14 (38.9%)
Yang [40]	1	1	0	0	3	1	0	0	1	0	0	2	0	0	0	0	9 (25.0%)
Yao [41]	1	1	0	0	3	1	0	1	2	0	0	2	0	0	0	0	11 (30.6%)
Zhang [42]	1	0	0	0	3	1	1	0	2	0	0	-5	0	2	0	0	5 (13.9%)
Zhang [43]	1	0	0	0	3	1	0	0	2	0	0	-5	0	2	0	0	4 (11.1%)
Zhang [44]	1	1	0	0	3	1	0	0	2	2	0	2	0	2	0	0	14 (38.9%)
Zhang [45]	1	1	0	0	3	1	1	1	2	2	0	-5	0	2	0	0	9 (25.0%)
Zhao [46]	0	1	0	0	3	0	0	1	2	2	0	-5	0	2	0	0	6 (16.7%)
Zhao [47]	1	1	0	0	3	1	0	1	2	2	0	2	0	2	0	0	15 (41.7%)
Zhao [48]	1	1	0	0	3	1	1	0	2	0	0	-5	0	2	0	0	6 (16.7%)
Zhou [49]	1	1	0	0	3	0	0	0	2	1	0	2	0	2	0	0	12 (33.3%)
Zhu [50]	0	1	0	0	3	0	1	0	2	0	0	2	0	2	0	0	11 (30.6%)

Zhu [51]	1	1	0	0	3	1	0	1	2	2	0	-5	0	2	0	1	9 (25.0%)
